# Cross-lagged panel analysis of kinesiophobia and frailty in older patients undergoing radical prostatectomy

**DOI:** 10.3389/fonc.2026.1914051

**Published:** 2026-10-12

**Authors:** Yue Gu, Hang Liu, TingYao Nie, YaNan Guo, Rui Yang, Ying Li, KeXin Zhao

**Affiliations:** 1 Department of Urology, The First Affiliated Hospital of Zhengzhou University, Zhengzhou, China; 2 Xiangya School of Nursing, Central South University, Changsha, China; 3 Department of Thoracic Surgery, The First Affiliated Hospital of Zhengzhou University, Zhengzhou, China

**Keywords:** cross-lagged panel study, frailty, kinesiophobia, older patients, radical prostatectomy

## Abstract

**Background:**

Kinesiophobia and frailty are common concerns during postoperative recovery in older adults, but their longitudinal relationship after radical prostatectomy (RP) remains unclear. This study examined changes in kinesiophobia and frailty and their reciprocal prospective associations during the first 3 months after RP.

**Methods:**

A prospective longitudinal study included 380 patients aged ≥65 years undergoing RP. Kinesiophobia and frailty were assessed at 24–48 hours, 1 month, and 3 months postoperatively using the TSK-17 and TFI. Cross-lagged panel models were estimated using robust maximum likelihood with full information maximum likelihood for missing data and adjustment for prespecified covariates. Complete-case analysis was performed as a sensitivity analysis.

**Results:**

Kinesiophobia and frailty both decreased significantly over the 3-month follow-up (both *P* < 0.001). T1 frailty was prospectively associated with T2 kinesiophobia (*β* = 0.210, 95% CI 0.121–0.300, *P* < 0.001), whereas T1 kinesiophobia was not associated with T2 frailty (*β* = 0.068, 95% CI −0.034–0.170, *P* = 0.193). From T2 to T3, significant prospective associations were observed in both directions: kinesiophobia with subsequent frailty (*β* = 0.278, 95% CI 0.181–0.374, *P* < 0.001) and frailty with subsequent kinesiophobia (*β* = 0.154, 95% CI 0.069–0.239, *P* < 0.001).

**Conclusions:**

Kinesiophobia and frailty were dynamically related during early recovery after RP, with their prospective associations varying across postoperative stages. These findings highlight the importance of considering both constructs over time and may help inform stage-specific monitoring during postoperative recovery.

## 1 Introduction

Prostate cancer is one of the most common malignancies among older men (1). In China, the average age at diagnosis is approximately 72 years (2). For patients with non-metastatic disease, radical prostatectomy (RP) is an important treatment option, but postoperative complications such as urinary incontinence and erectile dysfunction may adversely affect recovery and quality of life (3). Enhanced Recovery After Surgery (ERAS) programs for RP emphasize multimodal perioperative care, including early mobilization, and have been associated with faster postoperative recovery and shorter hospital stay (4, 5). Active participation in postoperative rehabilitation is therefore particularly important for older patients, in whom recovery may be further complicated by reduced physiological reserve and age-related functional limitations.

Kinesiophobia, defined as an excessive fear of movement or physical activity arising from a perceived vulnerability to pain or reinjury, may represent an important barrier to rehabilitation (6). Previous studies have reported a substantial prevalence of kinesiophobia in postoperative populations and have linked higher levels of kinesiophobia with poorer functional recovery and rehabilitation outcomes (7, 8). Following RP, postoperative discomfort, urinary leakage, and reduced physical function may complicate early recovery and participation in rehabilitation (4, 5, 9). This is particularly relevant during the early recovery period, when patients are expected to gradually resume daily activities and engage in postoperative rehabilitation. Fear-related avoidance of activity may be associated with lower rehabilitation participation, reduced activity levels, and poorer functional recovery (6, 8). Persistent movement-related fear may also be accompanied by negative expectations about physical activity and reduced confidence in engaging in movement safely (6). These concerns may be particularly relevant for older patients, whose postoperative recovery can be further challenged by reduced physiological reserve and age-related functional limitations. Therefore, identifying factors associated with postoperative kinesiophobia may be clinically important for supporting rehabilitation participation and functional recovery after RP.

Frailty is particularly relevant in this context because it reflects reduced physiological reserve and increased vulnerability to stressors in older adults (10). It is a multidimensional geriatric syndrome involving physical, psychological, and social deficits (11), and is common among older patients with cancer (12). Previous studies have shown that frailty is associated with a range of adverse postoperative outcomes, including prolonged hospitalization, postoperative complications, functional decline, impaired quality of life, and increased mortality (12–14). Importantly, frailty is not a static condition but may fluctuate over the course of postoperative recovery (15). In older patients undergoing RP, frailty may be particularly relevant during the early recovery period.

From the perspective of the fear-avoidance framework, fear of movement may promote avoidance or restriction of physical activity, thereby reducing participation in daily and rehabilitative activities (16, 17). Sustained reductions in activity may, in turn, be associated with physical deconditioning and greater functional vulnerability (16, 17). Conversely, frailty-related limitations, including fatigue, weakness, impaired balance, and reduced exercise tolerance, may increase the perceived difficulty or threat associated with movement and reduce confidence in engaging in physical activity, thereby reinforcing movement-related fear and avoidance (18). Together, these potential reciprocal processes provide a theoretical basis for examining whether earlier kinesiophobia is associated with subsequent frailty and whether earlier frailty is associated with subsequent kinesiophobia.

However, most previous studies examining the relationship between kinesiophobia and frailty have been cross-sectional, limiting the ability to clarify their temporal ordering (19, 20). Longitudinal evidence on the reciprocal associations between these two constructs remains limited, particularly among older patients undergoing RP. The cross-lagged panel model (CLPM) provides a useful framework for examining reciprocal longitudinal associations while accounting for the temporal stability of each construct (21). Although CLPM does not establish causality, it can help clarify whether earlier levels of one variable are associated with subsequent levels of the other over time (21). Accordingly, this prospective longitudinal study assessed kinesiophobia and frailty at 24–48 hours (T1), 1 month (T2), and 3 months (T3) after RP. The study aimed to examine changes in kinesiophobia and frailty during early postoperative recovery and to investigate their reciprocal longitudinal associations using a CLPM.

## 2 Methods

### 2.1 Study design

This study adopted a prospective longitudinal design and was reported in accordance with the Strengthening the Reporting of Observational Studies in Epidemiology (STROBE) statement. Three postoperative assessment time points were selected to capture clinically distinct phases of early recovery over a 3-month follow-up period. T1 was scheduled at 24–48 hours postoperatively to capture the immediate postoperative phase, when marked changes in physical function and activity patterns occur after surgery. T2 was scheduled at 1 month postoperatively to represent the early home-rehabilitation period, during which patients gradually resume daily activities and participate in postoperative rehabilitation. T3 was scheduled at 3 months postoperatively to reflect a later stage of short-term recovery, when functional recovery has progressed beyond the immediate postoperative phase.

### 2.2 Participants

Older patients with prostate cancer were recruited using convenience sampling from the Department of Urology of a tertiary Grade A general hospital in Henan Province, China, between March 2025 and March 2026. The inclusion criteria were as follows: (1) histopathologically confirmed prostate adenocarcinoma; (2) undergoing radical prostatectomy; and (3) age ≥65 years. The exclusion criteria were: (1) other malignant tumors or metastatic disease; (2) severe cognitive impairment or altered mental status that precluded completion of the study assessments; and (3) refusal to participate or withdrawal from the study. Sample size was estimated using a Monte Carlo simulation-based sensitivity analysis (22). Assuming a standardized cross-lagged effect of 0.15, a two-sided α of 0.05, and 5,000 replications, approximately 360 participants were required to achieve 80% power.

A follow-up team was established before study initiation and consisted of one urologist, two senior nurses, and two postgraduate nursing students. All team members completed standardized training on postoperative rehabilitation after RP, the definitions and assessment procedures for kinesiophobia and frailty, communication skills, and standardized telephone follow-up procedures. After training, an assessment was conducted to ensure consistency among team members in their understanding and administration of the study instruments. At T1, demographic and clinical information was collected, and kinesiophobia and frailty were assessed using the Tampa Scale for Kinesiophobia-17 (TSK-17) and the Tilburg Frailty Indicator (TFI), respectively, through on-site questionnaire administration and review of the electronic medical record system. At T2 and T3, the TSK-17 and TFI were administered by trained study personnel through standardized telephone interviews, each lasting approximately 15–20 minutes. Written informed consent was obtained from all participants before enrollment. For participants who were unable to complete the questionnaires independently, trained research personnel recorded their responses according to standardized procedures. During the data-collection period, the research team held weekly meetings to maintain procedural consistency and continuity of follow-up. After data collection was completed, two researchers independently entered the data and cross-checked all entries.

### 2.3 Measures

#### 2.3.1 Tampa scale for kinesiophobia-17

The Tampa Scale for Kinesiophobia-17 (TSK-17), which was developed by Kori et al. (23). and translated and validated into Chinese by Hu et al. (24)., was used to assess patients’ degree of fear of movement. This scale consists of 17 items scored on a 4-point Likert scale, with total scores ranging from 17 to 68. A score greater than 37 indicates clinically significant kinesiophobia; higher scores indicate a more severe fear of movement. The scale has demonstrated good reliability and validity, With a Cronbach’s α coefficient of 0.778. In the present study, the Cronbach’s α coefficient was 0.794.

#### 2.3.2 Tilburg frailty indicator

The Tilburg Frailty Indicator (TFI), developed by Gobbens et al (25). and translated into Chinese by Xi et al. (26), was used to assess the patients’ frailty status. This 15-item scale covers three domains: the physical domain (8 items), the psychological domain (4 items) and the social domain (3 items). Of these, 11 items are scored dichotomously and 4 items are scored trichotomously, yielding a total score ranging from 0 to 15. A total score of 5 or higher (≥ 5) indicates frailty, with higher scores indicating more severe frailty. In the Chinese validation study, the TFI showed a Cronbach’s α coefficient of 0.686. In the present study, the Cronbach’s α coefficient was 0.702.

#### 2.3.3 Age-adjusted charlson comorbidity index

The Charlson Comorbidity Index (CCI) was originally developed by Charlson et al. in 1987 to assess the burden of chronic comorbid conditions in patients, irrespective of the primary diagnosis (27). It comprises 17 disease categories, each assigned a weight of 1, 2, 3, or 6 points according to disease severity. The total score is calculated as the sum of the weighted scores, with higher scores indicating a greater comorbidity burden and a higher risk of adverse outcomes (27). An age-adjusted version, the Age-Adjusted Charlson Comorbidity Index (ACCI), was subsequently introduced in 1994 by incorporating age-specific weights: 1 point for patients aged 50–59 years, 2 points for those aged 60–69 years, 3 points for those aged 70–79 years, and 4 points for those aged ≥80 years (28). The ACCI was used in the present study. The total score was categorized into four levels: 0, no comorbidity; 1–2, mild; 3–4, moderate; and ≥5, severe comorbidity burden.

### 2.4 Statistical methods

Statistical analysis were performed using R version 4.3.3. Continuous variables with non-normal distributions are presented as medians and interquartile ranges [M (IQR)], and categorical variables as frequencies and percentages [n (%)]. To assess potential attrition bias, baseline demographic and clinical characteristics and T1 kinesiophobia and frailty scores were compared between participants who completed all three assessments and those lost to follow-up. Continuous variables were compared using the Mann–Whitney U test, and categorical variables using the chi-square test or Fisher’s exact test with Monte Carlo simulation, as appropriate. Among participants with complete data at all three time points, changes in kinesiophobia and frailty scores across T1, T2, and T3 were examined using the Friedman test, while changes in the prevalence of kinesiophobia and frailty were assessed using Cochran’s Q test; Bonferroni-adjusted pairwise comparisons were performed when appropriate. Spearman’s rank correlation analysis was used to examine within- and across-wave associations between kinesiophobia and frailty. All tests were two-sided, with P < 0.05 considered statistically significant.

Cross-lagged panel models were fitted using the lavaan package (29) to examine the longitudinal reciprocal associations between kinesiophobia and frailty. Covariates were selected *a priori* based on their clinical relevance and evidence from previous studies. The primary model included autoregressive, cross-lagged, and within-wave associations and was adjusted for ACCI, living arrangement, monthly income, Gleason score, and surgical approach. All 380 enrolled participants were included in the primary analysis. Based on the comparison of baseline demographic and clinical characteristics and T1 kinesiophobia and frailty scores between participants who completed all follow-up assessments and those lost to follow-up, the missing data were assumed to be Missing at Random (MAR). Missing longitudinal data were handled using full information maximum likelihood (FIML) (30). Robust maximum likelihood estimation was applied, and model fit was evaluated using scaled χ², χ²/df, robust CFI, robust TLI, robust RMSEA with 95% CI, and SRMR (31). Standardized coefficients (*β*), standard errors, 95% CIs, and P values were reported. The primary model was compared with three nested models in which all cross-lagged paths, kinesiophobia-to-frailty paths, or frailty-to-kinesiophobia paths were constrained to zero, respectively, using Satorra–Bentler scaled χ² difference tests (32). A complete-case analysis (*n* = 343) was additionally performed as a sensitivity analysis.

## 3 Results

### 3.1 Participant flow and baseline characteristics

A total of 380 patients with prostate cancer undergoing radical prostatectomy were enrolled at T1. During follow-up, 21 participants were lost at T2 and an additional 16 participants were lost at T3. Consequently, 359 participants completed the T2 assessment and 343 completed the T3 assessment, corresponding to an overall retention rate of 90.26%. No significant differences were observed in demographic and clinical characteristics or in T1 kinesiophobia and frailty between participants who completed all three assessments and those who did not complete follow-up (all P > 0.05). (**Supplementary Table S1**).

**Table 1** summarizes the demographic and clinical characteristics of the 380 participants. Of the participants, 215 (56.58%) were aged >70 years and 223 (58.68%) had an educational level of primary school or below. Most participants did not live alone (310, 81.58%). The New Rural Cooperative Medical Scheme was the most common type of health insurance (228, 60.00%), and 252 participants (66.32%) reported a monthly income of 2,000–4,000 RMB. In addition, 299 participants (78.68%) had an ACCI score of ≥5, and 210 (55.26%) had a Gleason score of 6–7. Laparoscopic surgery was performed in 163 participants (42.89%), whereas 217 participants (57.11%) underwent robot-assisted surgery.

**Table 1:** Demographic and clinical characteristics of the study participants (N = 380).

Variable	Category	n(%)
Age(years)	65-70	165 (43.42)
	>70	215 (56.58)
Educational level	primary school or below	223 (58.68)
	junior high school	74 (19.47)
	high school or technical secondary school	62 (16.32)
	college or above	21 (5.53)
Living arrangement	living alone	70 (18.42)
	not living alone	310 (81.58)
Type of health insurance	New Rural Cooperative Medical Scheme	228 (60.00)
	Urban Resident Basic Medical Insurance	84 (22.11)
	Urban Employee Basic Medical Insurance	56 (14.74)
	other	12(3.16)
Monthly income(RMB)	< 2,000	102 (26.84)
	2,000–4,000	252 (66.32)
	> 4,000	26 (6.84)
ACCI(points)	4	81 (21.32)
	≥5	299 (78.68)
Surgical approach(points)	Laparoscopic approaches	163 (42.89)
	robot-assisted	217 (57.11)
Gleason(points)	6–7	210 (55.26)
	≥8	170 (44.74)

### 3.2 Changes in kinesiophobia and frailty across follow-up

Among the 343 participants who completed all three assessments, both kinesiophobia and frailty decreased progressively over the 3-month postoperative follow-up period. The median kinesiophobia score decreased from 38 (37, 39) at T1 to 34 (32, 36) at T2 and 32 (30, 34) at T3. Similarly, the median frailty score decreased from 6 (5, 8) at T1 to 4 (3, 5) at T2 and 3 (3, 4) at T3. Friedman tests showed significant differences across the three assessments for both kinesiophobia (χ² = 636.61, P < 0.001) and frailty (χ² = 598.46, P < 0.001).

The prevalence of clinically significant kinesiophobia was 75.22% at T1 (95% CI: 70.39%–79.49%), 18.37% at T2 (95% CI: 14.63%–22.81%), and 0.58% at T3 (95% CI: 0.16%–2.10%). Frailty prevalence was 95.34% at T1 (95% CI: 92.56%–97.11%), 48.98% at T2 (95% CI: 43.73%–54.25%), and 18.08% at T3 (95% CI: 14.36%–22.49%). Cochran’s Q tests showed significant overall differences in the prevalence of both kinesiophobia and frailty across the three assessments (both P < 0.001). Bonferroni-adjusted pairwise comparisons further indicated significant decreases between all pairs of time points (all *P* < 0.001). (**Table 2**).

**Table 2:** Changes in kinesiophobia and frailty across the three postoperative assessments (n = 343).

Time point	Kinesiophobia		Frailty	
	*M* (IQR)	*n* (%)	*M* (IQR)	*n* (%)
T1	38 (37, 39)	258 (75.22)	6 (5, 8)	327 (95.34)
T2	34 (32, 36)	63 (18.37)	4 (3, 5)	168 (48.98)
T3	32 (30, 34)	2 (0.58)	3 (3, 4)	62 (18.08)
Statistic	636.61^a^	415.82^b^	598.46^a^	392.43^b^
*P* value	<0.001	<0.001	<0.001	<0.001

^a^ Friedman χ² test; ^b^ Cochran’s Q test.

### 3.3 Correlations between kinesiophobia and frailty

Among the 343 participants who completed all three assessments, Spearman correlation analysis showed significant positive correlations between kinesiophobia and frailty both within and across the three postoperative assessments. At the same assessment wave, kinesiophobia was positively correlated with frailty at T1 (*r* = 0.764), T2 (*r* = 0.655), and T3 (*r* = 0.666), with all correlations reaching statistical significance (all *P* < 0.001). Significant positive correlations were also observed across assessment waves. These findings supported further examination of the longitudinal associations between kinesiophobia and frailty using a cross-lagged panel model (**Table 3**).

**Table 3:** Correlation analysis of kinesiophobia and frailty (n = 343).

Variables	1	2	3	4	5	6
T1 Kinesiophobia	–					
T2 Kinesiophobia	0.737^***^	–				
T3 Kinesiophobia	0.611^***^	0.761^***^	–			
T1 Frailty	0.764^***^	0.677^***^	0.595^***^	–		
T2 Frailty	0.617^***^	0.655^***^	0.599^***^	0.738^***^	–	
T3 Frailty	0.610^***^	0.656^***^	0.666^***^	0.649^***^	0.662^***^	

Values are Spearman’s rank correlation coefficients. ^***^P < 0.001.

### 3.4 Primary cross-lagged panel model

The primary cross-lagged panel model was estimated using all 380 enrolled participants, with all available longitudinal observations incorporated in the analysis. After adjustment for the prespecified covariates (ACCI, living arrangement, monthly income, Gleason score, and surgical approach), the primary cross-lagged panel model showed mixed fit indices (scaled χ² = 16.53, df = 4, χ²/df = 4.13, robust CFI = 0.992, robust TLI = 0.908, robust RMSEA = 0.098, 95% CI: 0.053–0.148, and SRMR = 0.012).

Significant autoregressive associations were observed for both constructs. Frailty at T1 was positively associated with frailty at T2 (*β* = 0.616, SE = 0.041, 95% CI: 0.536–0.696, *P* < 0.001), and frailty at T2 was positively associated with frailty at T3 (β = 0.377, SE = 0.052, 95% CI: 0.274–0.479, P < 0.001). Similarly, kinesiophobia showed significant temporal stability from T1 to T2 (*β* = 0.441, SE = 0.056, 95% CI: 0.331–0.550, *P* < 0.001) and from T2 to T3 (*β* = 0.573, SE = 0.048, 95% CI: 0.480–0.667, *P* < 0.001). Significant positive within-wave associations between frailty and kinesiophobia were observed at T1 (*β* = 0.571, SE = 0.036, 95% CI: 0.501–0.641, *P* < 0.001), T2 (*β* = 0.271, SE = 0.053, 95% CI: 0.166–0.375, *P* < 0.001), and T3 (*β* = 0.233, SE = 0.049, 95% CI: 0.137–0.329, *P* < 0.001).

Regarding the cross-lagged associations, T1 frailty was significantly associated with subsequent T2 kinesiophobia (*β* = 0.210, SE = 0.045, 95% CI: 0.121–0.300, *P* < 0.001), whereas T1 kinesiophobia was not significantly associated with subsequent T2 frailty (*β* = 0.068, SE = 0.052, 95% CI: −0.034–0.170, *P* = 0.193). During the later interval, both cross-lagged pathways were significant: T2 kinesiophobia was significantly associated with T3 frailty (*β* = 0.278, SE = 0.049, 95% CI: 0.181–0.374, *P* < 0.001), and T2 frailty was significantly associated with T3 kinesiophobia (*β* = 0.154, SE = 0.043, 95% CI: 0.069–0.239, *P* < 0.001) (**Table 4**, **Figure 1**).

**Figure 1:**
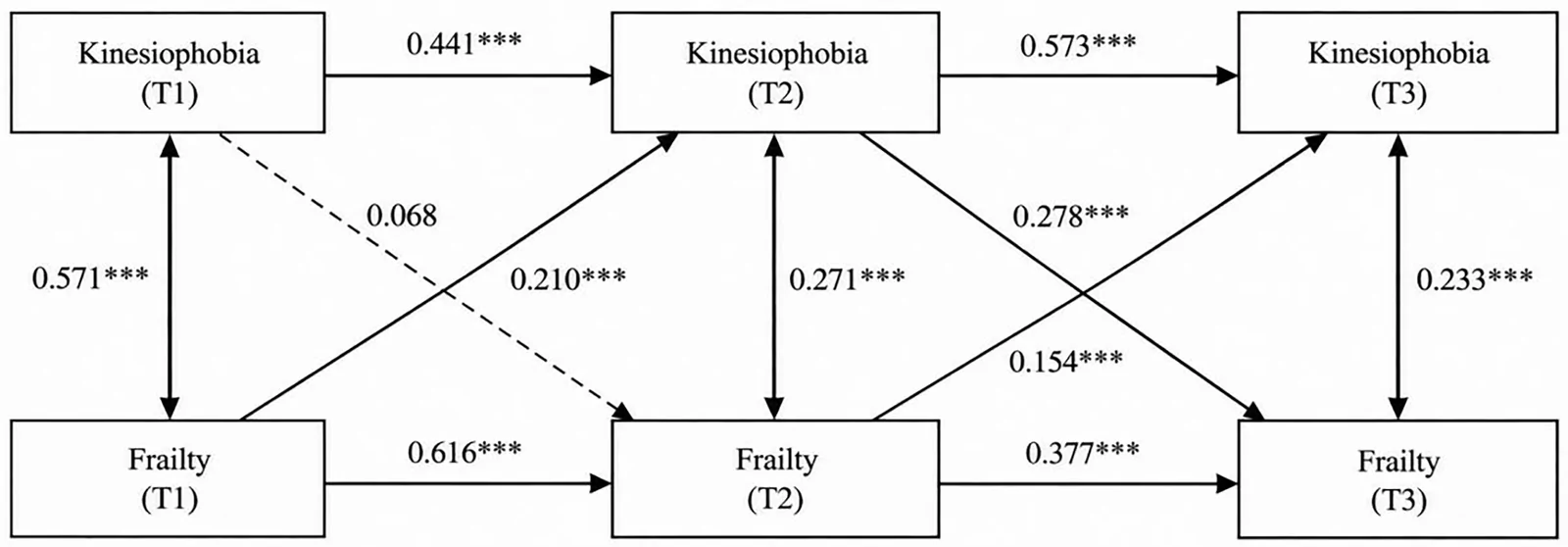
Primary cross-lagged panel model of kinesiophobia and frailty (N = 380).

**Table 4:** Parameter estimates of the primary cross-lagged panel model (*N* = 380).

Path type	Path	*β*	SE	95% CI	*P*
Autoregressive	T1 frailty → T2 frailty	0.616	0.041	0.536–0.696	<0.001
	T2 frailty → T3 frailty	0.377	0.052	0.274–0.479	<0.001
	T1 kinesiophobia → T2 kinesiophobia	0.441	0.056	0.331–0.550	<0.001
	T2 kinesiophobia → T3 kinesiophobia	0.573	0.048	0.480–0.667	<0.001
Cross-lagged	T1 kinesiophobia → T2 frailty	0.068	0.052	−0.034–0.170	0.193
	T1 frailty → T2 kinesiophobia	0.210	0.045	0.121–0.300	<0.001
	T2 kinesiophobia → T3 frailty	0.278	0.049	0.181–0.374	<0.001
	T2 frailty → T3 kinesiophobia	0.154	0.043	0.069–0.239	<0.001
Within-wave	T1 frailty ↔ T1 kinesiophobia	0.571	0.036	0.501–0.641	<0.001
	T2 frailty ↔ T2 kinesiophobia	0.271	0.053	0.166–0.375	<0.001
	T3 frailty ↔ T3 kinesiophobia	0.233	0.049	0.137–0.329	<0.001

*B*, standardized coefficient; SE, standard error; CI, confidence interval.

### 3.5 Model comparison

To evaluate the contribution of the cross-lagged pathways, the primary CLPM was compared with three constrained models. When all cross-lagged pathways were constrained to zero, model fit deteriorated substantially (robust CFI = 0.952, robust TLI = 0.728, robust RMSEA = 0.168, SRMR = 0.050), and the scaled chi-square difference test indicated a significantly poorer fit than the primary model (Δχ² = 71.075, Δdf = 4, *P* < 0.001). Similarly, constraining the pathways from kinesiophobia to subsequent frailty to zero resulted in poorer model fit (robust CFI = 0.973, robust TLI = 0.801, robust RMSEA = 0.144, SRMR = 0.029) and a significant deterioration relative to the primary model (Δχ² = 32.117, Δdf = 2, *P* < 0.001), while constraining the pathways from frailty to subsequent kinesiophobia to zero also worsened model fit (robust CFI = 0.974, robust TLI = 0.808, robust RMSEA = 0.142, SRMR = 0.032; Δχ² = 31.015, Δdf = 2, *P* < 0.001). These findings indicated that retaining the cross-lagged pathways provided a better representation of the longitudinal associations between kinesiophobia and frailty (**Table 5**).

**Table 5:** Comparison of fit indices for the primary and constrained cross-lagged panel models (N = 380).

Model	χ²	df	CFI	TLI	RMSEA	SRMR	Δχ²	Δdf	*P*
Primary CLPM	16.531	4	0.992	0.908	0.098	0.012	—	—	—
No cross-lagged paths	87.771	8	0.952	0.728	0.168	0.050	71.075	4	<0.001
Kinesiophobia → frailty paths constrained to zero	48.481	6	0.973	0.801	0.144	0.029	32.117	2	<0.001
Frailty → kinesiophobia paths constrained to zero	47.036	6	0.974	0.808	0.142	0.032	31.015	2	<0.001

CFI, comparative fit index; TLI, Tucker–Lewis index; RMSEA, root mean square error of approximation; SRMR, standardized root mean square residual. Robust fit indices were reported. Δχ², Δdf, and P values were obtained from Satorra–Bentler scaled chi-square difference tests comparing models.

### 3.6 Sensitivity analysis

To assess the robustness of the primary findings to the handling of missing data, the CLPM was re-estimated using complete cases only (*n* = 343). T1 kinesiophobia was not significantly associated with T2 frailty (*β* = 0.066, SE = 0.052, 95% CI: −0.037–0.168, *P* = 0.209), whereas T1 frailty was significantly associated with T2 kinesiophobia (*β* = 0.222, SE = 0.046, 95% CI: 0.131–0.312, *P* < 0.001). During the T2-to-T3 interval, both cross-lagged associations remained significant, with T2 kinesiophobia associated with subsequent T3 frailty (*β* = 0.283, SE = 0.049, 95% CI: 0.186–0.379, *P* < 0.001) and T2 frailty associated with subsequent T3 kinesiophobia (*β* = 0.150, SE = 0.043, 95% CI: 0.065–0.235, *P* < 0.001). Overall, the complete-case analysis yielded a pattern of cross-lagged associations similar to that observed in the primary analysis. Detailed results are presented in **Supplementary Table S2** and **Supplementary Figure S1**.

## 4 Discussion

This prospective longitudinal study identified distinct temporal patterns in the associations between kinesiophobia and frailty during the first 3 months after radical prostatectomy in older patients. Both constructs decreased progressively over time but remained temporally stable and concurrently associated across follow-up. After accounting for prespecified covariates, frailty at 24–48 hours was prospectively associated with greater kinesiophobia at 1 month, whereas the reverse association was not significant. In contrast, from 1 to 3 months, significant cross-lagged associations emerged in both directions. These findings indicate that the pattern of prospective associations between kinesiophobia and frailty differs across postoperative intervals and may evolve as recovery progresses. The complete-case sensitivity analysis yielded a similar pattern of cross-lagged associations, supporting the robustness of the primary findings to the handling of missing data.

Both kinesiophobia and frailty declined progressively at the group level during the first 3 months after RP. The relatively high level of kinesiophobia shortly after surgery could reflect a combination of surgical stress, postoperative discomfort, reduced mobility, fatigue, urinary symptoms, and uncertainty regarding the safety of physical activity (4–6, 9). As postoperative symptoms improve and patients gradually resume daily activities and become more familiar with rehabilitation, concerns about movement may lessen and confidence in physical activity may increase (6, 9). A similar recovery process could accompany the reduction in frailty. Because frailty reflects vulnerability across physical, psychological, and social domains (11, 25), reduced activity tolerance, poor appetite, sleep disturbance, fatigue, and temporary dependence during the immediate postoperative period could coincide with higher frailty levels. As mobility, endurance, nutritional intake, and participation in daily life gradually recover, overall frailty scores may subsequently decrease. This downward trend is broadly consistent with previous longitudinal evidence in older patients undergoing RP, although substantial heterogeneity in individual frailty trajectories has been reported (15). Accordingly, the observed decline should be interpreted as a group-level recovery trend rather than a uniform trajectory across all patients.

The significant autoregressive and concurrent relationships indicated that both kinesiophobia and frailty exhibited temporal stability and remained positively related throughout early postoperative recovery. More importantly, the cross-lagged findings differed by postoperative interval. From 24–48 hours to 1 month, greater frailty was prospectively linked to higher subsequent kinesiophobia, whereas the reverse pathway was not significant. From 1 to 3 months, however, significant prospective relationships were evident in both directions. The model-comparison results further supported retaining the cross-lagged pathways, as models in which these pathways were constrained to zero showed significantly poorer fit than the primary model. The asymmetry observed during the earlier interval could reflect the distinct context of the immediate postoperative period. At this stage, frailty may represent a combination of pre-existing vulnerability and transient postoperative reductions in physiological reserve (10, 12, 14). Frailty-related weakness, fatigue, impaired balance, and reduced exercise tolerance may increase the perceived effort and uncertainty associated with movement, making physical activity feel more difficult or potentially unsafe. Such functional limitations could, in turn, coincide with lower confidence in physical activity and greater movement-related concern, providing one possible physiological–behavioral explanation for the prospective association between early frailty and subsequent kinesiophobia (18–20). In contrast, kinesiophobia assessed within 24–48 hours may partly reflect acute postoperative concerns, and its relationship with subsequent frailty may not yet be evident during this early interval.

By 1 month after surgery, the recovery context may differ as patients increasingly resume daily activities and engage in home-based rehabilitation (9). Under these circumstances, a more reciprocal prospective relationship between kinesiophobia and frailty may become apparent. Persistent kinesiophobia could coincide with lower levels of physical activity and rehabilitation participation, factors that may also be relevant to later frailty (6, 8, 16, 17). Likewise, sustained movement avoidance could accompany lower rehabilitation participation, slower resumption of daily activities, greater physical deconditioning, reduced endurance, and lower participation in social and functional activities (8, 16, 17). From a physiological perspective, sustained reductions in physical activity may contribute to losses in muscle mass, strength, and functional capacity through disuse-related anabolic resistance and impaired muscle protein synthesis (33–35). These effects may be particularly relevant in older adults, who are more susceptible to inactivity-related reductions in skeletal muscle function and exercise capacity (33, 34). Such physiological changes could provide one plausible pathway linking persistent movement avoidance with greater subsequent physical vulnerability. Because frailty in this study was assessed using the multidimensional TFI, however, this pathway should not be interpreted as suggesting that frailty is solely a consequence of physical deconditioning; activity-related limitations may also extend to psychological and social dimensions of frailty (25, 26). Conversely, residual frailty at 1 month could remain linked to subsequent kinesiophobia, potentially in the context of persistent fatigue, weakness, instability, and reduced confidence in engaging in activity safely (19, 20). Repeated difficulty during physical activity may also coincide with more negative expectations about movement and greater avoidance (16, 17). Taken together, these findings are consistent with a reciprocal prospective relationship between kinesiophobia and frailty during the later phase of early recovery.

The changing prospective relationships across postoperative intervals suggest that the clinical significance of frailty and kinesiophobia may vary over the course of early recovery. During the immediate postoperative period, the high prevalence of kinesiophobia underscores the clinical importance of identifying movement-related fear, while early frailty may offer additional information because of its prospective link with subsequent kinesiophobia. Assessment of frailty-related features such as mobility limitation, fatigue, balance impairment, and reduced exercise tolerance could therefore help identify patients who warrant closer monitoring for persistent movement-related fear during early recovery. From 1 to 3 months after surgery, when significant prospective relationships were evident in both directions, joint assessment of frailty and kinesiophobia may be particularly informative. These findings may help define clinically meaningful targets and time windows for future rehabilitation research. However, intervention studies are still needed to determine whether targeting either construct at specific time points improves subsequent recovery outcomes.

## 5 Limitations

Several limitations should be considered when interpreting the findings of this study. First, participants were recruited using convenience sampling from a single tertiary hospital in China, which may limit the generalizability of the findings to older patients undergoing radical prostatectomy in other healthcare settings or populations. Second, kinesiophobia and frailty were first assessed at 24–48 hours after surgery rather than preoperatively. Therefore, the T1 frailty score may reflect both pre-existing vulnerability and transient responses to acute surgical stress. Third, although the traditional cross-lagged panel model allowed examination of prospective reciprocal associations while accounting for prior levels of each construct, it cannot fully separate stable between-person differences from within-person changes over time. Therefore, some cross-lagged associations may partly reflect stable individual differences. Fourth, although the models were adjusted for several clinically relevant covariates, residual confounding cannot be excluded because postoperative pain, physical activity, nutritional status, social support, and postoperative complications were not directly incorporated into the analyses. Fifth, the mode of assessment differed across follow-up, with face-to-face assessment at T1 and telephone interviews at T2 and T3, which may have introduced mode-related measurement differences. In addition, although the TSK-17 and TFI are validated instruments, the clinical meaning of established cut-off values, particularly for kinesiophobia, may differ in older patients recovering from RP. Finally, although the primary model showed favorable CFI and SRMR values, the RMSEA indicated less optimal fit, suggesting some inconsistency across model-fit indices. Therefore, the cross-lagged estimates should be interpreted with appropriate caution. Future multicenter studies incorporating preoperative assessments, more evenly spaced follow-up intervals, repeated measures of relevant postoperative factors, and alternative longitudinal models may help further clarify the temporal relationship between kinesiophobia and frailty.

## 6 Conclusion

Kinesiophobia and frailty decreased during the first 3 months after radical prostatectomy in older patients, while the pattern of their prospective associations differed across postoperative intervals. Frailty at 24–48 hours was associated with subsequent kinesiophobia at 1 month, whereas the reverse association was not significant. From 1 to 3 months, significant prospective associations were observed in both directions between kinesiophobia and frailty. These findings suggest that the temporal relationship between frailty and kinesiophobia may vary across the course of early postoperative recovery and support the potential value of monitoring both constructs during follow-up. Further longitudinal studies are needed to confirm these temporal patterns and clarify their clinical implications.

## Data Availability

The raw data supporting the conclusions of this article will be made available by the authors, without undue reservation.
