# Supplementary material for: Cross-lagged panel analysis of kinesiophobia and frailty in older patients undergoing radical prostatectomy
Source: Front Oncol. 2026 Oct 12;16:1914051. doi: 10.3389/fonc.2026.1914051 (PMC13696203; doi:10.3389/fonc.2026.1914051)
Supplement: Supplementary file 1 [file DataSheet1.docx]

**Supplementary Table S1.** Comparison of baseline characteristics between participants who completed all three assessments and those lost to follow-up

| **Variable** | **Category** | **Completers**  **(*n* = 343)** | **Lost to follow-up**  **(*n* = 37)** | **Test statistic** | ***P*** |
| --- | --- | --- | --- | --- | --- |
| Age (years) | 65-70 | 150 (43.73) | 15 (40.54) | 0.138 | 0.710^a^ |
|  | >70 | 193 (56.27) | 22 (59.46) |  |  |
| Educational level | primary school or below | 191 (55.69) | 14 (37.84) | - | 0.169^b^ |
|  | junior high school | 69 (20.12) | 10 (27.03) |  |  |
|  | high school or technical secondary school | 62 (18.08) | 10 (27.03) |  |  |
|  | college or above | 21 (6.12) | 3 (8.11) |  |  |
| Living arrangement | living alone | 59 (17.20) | 11 (29.73) | 3.488 | 0.062^a^ |
|  | not living alone | 284 (82.80) | 26 (70.27) |  |  |
| Type of health insurance | New Rural Cooperative Medical Scheme | 196 (57.14) | 18 (48.65) | - | 0.409^b^ |
|  | Urban Resident Basic Medical Insurance | 79 (23.03) | 10 (27.03) |  |  |
|  | Urban Employee Basic Medical Insurance | 56 (16.33) | 9 (24.32) |  |  |
|  | other | 12 (3.50) | 0 (0.00) |  |  |
| Monthly income (RMB) | < 2,000 | 89 (25.95) | 13 (35.14) | - | 0.139^b^ |
|  | 2,000–4,000 | 228 (66.47) | 24 (64.86) |  |  |
|  | > 4,000 | 26 (7.58) | 0 (0.00) |  |  |
| ACCI (points) | 4 | 77 (22.45) | 4 (10.81) | 2.697 | 0.101^a^ |
|  | ≥5 | 266 (77.55) | 33 (89.19) |  |  |
| Surgical approach (points) | Laparoscopic approaches | 157 (45.77) | 16 (43.24) | 0.086 | 0.769^a^ |
|  | robot-assisted | 186 (54.23) | 21 (56.76) |  |  |
| Gleason (points) | 6–7 | 185 (53.94) | 25 (67.57) | 2.510 | 0.113^a^ |
|  | ≥8 | 158 (46.06) | 12 (32.43) |  |  |
| T1 kinesiophobia score, M (IQR) |  | 38 (37, 39) | 39 (37, 39) | 5130.5 | 0.052^c^ |
| T1 frailty score, M (IQR) |  | 6 (5, 8) | 6 (5, 8) | 6319.0 | 0.967^c^ |

**Note:**  ^a^  Pearson’s chi-square test; ^b^ Fisher’s exact test with Monte Carlo simulation; ^c^ Mann–Whitney U test.

**Supplementary Table S2.** Parameter estimates of the complete-case cross-lagged panel model (*n* = 343)

| **Path type** | **Path** | ***β*** | **SE** | **95% CI** | ***P*** |
| --- | --- | --- | --- | --- | --- |
| Autoregressive | T1 frailty → T2 frailty | 0.633 | 0.041 | 0.553–0.714 | <0.001 |
|  | T2 frailty → T3 frailty | 0.368 | 0.051 | 0.268–0.468 | <0.001 |
|  | T1 kinesiophobia → T2 kinesiophobia | 0.438 | 0.057 | 0.327–0.549 | <0.001 |
|  | T2 kinesiophobia → T3 kinesiophobia | 0.578 | 0.048 | 0.484–0.672 | <0.001 |
| Cross-lagged | T1 kinesiophobia → T2 frailty | 0.066 | 0.052 | −0.037–0.168 | 0.209 |
|  | T1 frailty → T2 kinesiophobia | 0.222 | 0.046 | 0.131–0.312 | <0.001 |
|  | T2 kinesiophobia → T3 frailty | 0.283 | 0.049 | 0.186–0.379 | <0.001 |
|  | T2 frailty → T3 kinesiophobia | 0.150 | 0.043 | 0.065–0.235 | <0.001 |
| Within-wave | T1 frailty ↔ T1 kinesiophobia | 0.602 | 0.034 | 0.535–0.669 | <0.001 |
|  | T2 frailty ↔ T2 kinesiophobia | 0.255 | 0.056 | 0.144–0.365 | <0.001 |
|  | T3 frailty ↔ T3 kinesiophobia | 0.233 | 0.049 | 0.137–0.329 | <0.001 |

**Note:** β = standardized coefficient; SE = standard error; CI = confidence interval.

**Supplementary Figure S1.** Complete-case cross-lagged panel model of kinesiophobia and frailty (*n* = 343)
